# Merge-YOLO: An accurate detection model for book packaging defects in intelligent logistics scenarios

**DOI:** 10.1371/journal.pone.0340205

**Published:** 2026-01-08

**Authors:** Zhaohua Wang, Yirong Luo, Yanping Du, Jiawen Li, Yuqian Wang, Yuhao Lin

**Affiliations:** 1 School of Mechanical and Electrical Engineering, Beijing Institute of Graphic Communication, Beijing, People’s Republic of China; 2 Beijing Institute of Graphic Communication Comprehensive Laboratory for Key Technology Research and Application in the Field of News and Publishing, Beijing, People’s Republic of China; Yangtze University, CHINA

## Abstract

Driven by the knowledge economy and digitalization, the scale of book logistics continues to expand. However, the quality inspection process in this field currently uses generic target detection models and rarely considers defect characteristics. Therefore, this paper proposes the Merge-YOLO model to address the three prominent characteristics of book packaging defects: low contrast, small-sized defects, and irregular shapes. Three improvements are made to enhance detection performance: the WT-C3k2 module is designed to separate high- and low-frequency features using wavelet transforms, combined with multi-level convolutions and a bottleneck structure to enhance feature extraction capabilities for small objects and complex lighting conditions, while expanding the receptive field and reducing semantic detail loss; introducing the QA Transformer, which uses a learnable transformation matrix to generate adaptive quadrilateral windows, breaking through the limitations of traditional fixed windows and improving the ability to capture features of irregular defects; and adopting the DySample dynamic upscaler, which replaces nearest-neighbor interpolation by dynamically adjusting the scaling ratio through an adaptive scope factor, reducing computational overhead while preserving pixel-level details. Experiments show that the model achieves 95.8% precision, 93.6% recall, and 94.1%mAP@0.5 on the book packaging defect dataset, outperforming the baseline model YOLOv11 and traditional algorithms in all metrics. This provides an efficient and accurate detection model for quality control in book supply chain packaging.

## 1. Introduction

The rapid expansion of online book purchasing platforms, along with the transformation and innovation of physical bookstores, has led to continuous growth in book logistics. However, books are fragile items, and their packaging logistics are characterized by high frequency, diverse product categories, and susceptibility to damage. This is particularly true for paper publications and cultural and creative products, which impose stringent requirements on packaging quality.

Currently, the logistics packaging systems used by bookstores face three major challenges:

First, conventional packaging materials such as corrugated cardboard boxes and plastic film fail to provide adequate protection against compression, moisture, and folding, resulting in a high incidence of damage—including torn covers and creased pages.

Second, packaging defects are diverse in nature. These include surface damage (such as tearing or punctures), inadequate sealing (e.g., poorly adhered tape or cracked adhesive), and structural deformation (such as collapsed internal supports or cracked edges). Such defects frequently lead to book damage or loss across various scenarios, including online retail, warehouse distribution for chain bookstores, and cross-regional inventory transfers.

Third, existing quality inspection processes are insufficient. Current methods suffer from low efficiency and a high rate of undetected defects, failing to meet the precision and throughput demands of modern logistics operations.

To address the aforementioned challenges in packaging defect detection, this paper focuses on three key characteristics of book packaging—low defect contrast, small defect size, and irregular defect shapes—to enhance detection performance. Low-contrast defects, such as superficial scratches and minor wrinkles, often result from material reflectivity, uneven lighting, or background texture interference, leading to minimal grayscale or color differences between defective and non-defective regions. Current defect detection methods can be broadly categorized into three types: manual inspection, traditional object detection, and deep learning-based object detection. Manual inspection relies heavily on the expertise of quality control personnel and is susceptible to subjective factors such as lighting conditions and inspector fatigue, resulting in low efficiency and high missed-detection rates. Traditional object detection methods depend on handcrafted features, which struggle to capture subtle variations in complex defects like irregular damage and shallow scratches, and exhibit limited generalization capability. Although deep learning-based object detection methods have improved precision, some models suffer from high computational complexity and poor real-time performance. Moreover, lightweight one-stage models often face challenges in extracting sufficient features for detecting small defects and identifying low-contrast anomalies.

In the following section, we review two main detection approaches: traditional object detection methods and deep learning-based object detection methods.

## 2. Literature review

Traditional object detection methods rely on manually designed feature extractors and classifiers (Maoqing C et al. [1], Dash P.K et al. [2], Yang Xi et al. [3]), which have been applied to logistics packaging defect detection to a certain extent. For example, early studies used edge detection and morphological analysis to identify tears or indentations on the surface of cardboard boxes, but such methods have significant limitations: first, artificially designed features are unable to effectively capture the subtle differences in complex defects (such as irregular damage or shallow scratches) and are sensitive to environmental changes such as lighting and angle; Second, traditional methods have limited generalization capabilities, only applicable to specific types or high-contrast defects, and cannot adapt to the diverse product categories and scenarios required for book packaging inspection. Finally, they rely on manual parameter tuning and multi-stage processing, resulting in low efficiency and inability to meet the real-time inspection requirements of modern logistics.

As industrial production demands ever-increasing precision and efficiency in inspection, vision-language interaction models [4] and various defect detection models for industrial inspection have now emerged [5–8].

As a mainstream technology in the field of object detection, deep learning significantly enhances detection precision and efficiency through its powerful automatic feature extraction capabilities. By leveraging extensive datasets and neural network architectures, deep learning models autonomously extract complex features and patterns from images, outperforming traditional methods.

Deep learning-based object detection algorithms are mainly divided into two-stage object detection algorithms and one-stage object detection algorithms. The two-stage object detection algorithm achieves high-precision detection through two stages: “generating candidate target areas + classification and localization.” Many scholars have used various improvement methods to enhance detection precision. For example, by integrating the advantages of two different convolutional neural networks, detection precision and localization precision can be improved (Zhang X et al. [9] combined Faster R-CNN with R-FCN, significantly improving target recall and precision; Gao Boxuan et al. [10] propose a multi-model cascade framework that generates high-quality defect samples through an improved generative adversarial network and improves classification performance through an optimized VGG network.); improve the detection precision of the model by expanding the image dataset or using sample datasets with monochrome backgrounds (Zhao Y et al. [11] constructed a light-emitting diode defect database for photovoltaic modules and used a trained convolutional neural network to achieve high-precision defect identification for additional collected defect data; Zhai Yongjie et al. [12] proposed a geometric feature learning region convolutional neural network (GCL R-CNN) that improves the precision of vibration damper defect detection in overhead images of power transmission lines by introducing a GCL module and generating artificial samples in Faster R-CNN.); improving detection precision through multi-scale feature fusion(Wu X et al. [13] proposed a multi-scale region proposal network (MS-RPN) based on the Faster R-CNN framework and a positive sample adaptive loss function (PSALF), achieving a detection precision of 99.03% in open pinhole defect detection; Hao R et al. [14] designed a backbone network based on deformable convolution enhancement to adapt to different defect shapes, achieving an average precision of 80.5% in steel defect detection.); achieving high-precision detection through the introduction of attention mechanisms (Meng D et al. [15] Optimized pixel-level defect detection on aircraft surfaces based on Mask R-CNN through attention mechanisms and feature fusion modules; Wang X et al. [16] introduced an efficient channel attention mechanism after the feature extraction stage of Faster R-CNN, significantly improving the model’s feature representation performance in identifying small defects.).

Although two-stage object detection algorithms achieve high precision, their computational demands and slower inference speeds—stemming from the two independent processing stages—often hinder their applicability in real-time scenarios such as modern logistics. To mitigate these limitations, one-stage object detection algorithms have been introduced, prioritizing detection speed through an end-to-end prediction approach.

In this paradigm, object detection is treated as a regression problem, with the model directly predicting both the category and location of objects in the image. While this design enables significantly faster inference, it generally comes at a slight cost to precision compared to two-stage methods. Consequently, a key focus of recent research has been on enhancing the precision of one-stage detectors without compromising their speed advantages.

Improve detection precision by designing an efficient feature fusion module (Cheng Xun et al. [17] proposed a RetinaNet with different channel attention and adaptive spatial feature fusion, which improves detection precision through anchor optimization using differential evolution search, achieving a 2.92% improvement in average precision on the steel surface defect dataset.); improving detection precision by combining the advantages of two algorithms (Yongming Han et al. [18] combined SSD with optical flow and improved precision by 2.93% and reduced error rate by 25.4% on a boiler waterwall surface defect dataset using optical flow and reaction force.); by adopting an attention mechanism, it adapts to different types of defects (Lu Bingyu et al. [19] designed an adaptive feature fusion module to fuse and enhance multi-resolution features, using an attention mechanism to make the feature fusion process adaptive to input fabric images with different textures. It improved the mAP metric by 3.7% compared to other two-stage models and by 9.3% compared to one-stage detection models.); there is also a detection model that combines the above two methods (Hebbache Loucif et al. [20] proposed an efficient one-stage detection model, SMDD-Net, which combines pyramid feature extraction and attention mechanisms to achieve automatic detection of concrete defects in bridges from drone images, achieving an average precision of up to 99.1%.).

A more common approach is to improve detection precision in the YOLO series through data augmentation (Iqra et al. [21], Seung Ju Lee et al. [22], Lin J et al. [23], Li S et al. [24]), network architecture optimization (G. B. Rajendran et al. [25], Wu Y et al. [26], Yang Y et al. [27], Hou H et al. [28]), introduction of attention mechanisms (Chen J et al. [29], Cheng A et al. [30], Qiming Z et al. [31]), improvement of loss functions (Ma C et al. [32], Lu J et al. [33], Tang C et al. [34], Lin F et al. [35]), and multi-scale feature fusion strategies Zexuan G et al. [36], Tong Y et al. [37], Fu X et al. [38], Tianyu W et al. [39]).

Overall, existing detection methods face two major challenges in the context of book logistics packaging: first, poor environmental robustness, as uneven lighting (strong light, backlighting) in warehouses can lead to unstable feature extraction, while manual detection is subject to subjective factors. Secondly, it is difficult to achieve a balance between efficiency and precision. Complex models are computationally expensive and cannot meet real-time detection requirements, while lightweight models sacrifice some precision in detection.

For the scenario of defect detection in bookstore logistics packaging, we proposed the Merge-YOLO model, which optimizes the one-stage object detection model to address the bottlenecks of traditional methods in terms of precision, efficiency, and environmental adaptability. This model solves inherent problems in surface defect detection, including wide variations in defect size, low contrast between defects and the background, and the need for high detection speed and precision. Providing innovative solutions for quality control of packaging in the bookstore supply chain, the main contributions of this paper are as follows:

1) We propose a feature extraction module based on wavelet transformation, WT-C3k2, which effectively increases the receptive field using wavelet transformation to accurately identify packaging defect images collected in different storage environments.
2) To improve the precision of detecting irregular defects, we introduced QA Transformer to generate adaptive quadrilateral windows, enhancing the ability to capture features of arbitrarily shaped targets. We also replaced traditional interpolation methods with DySample dynamic upsamplers, reducing computational overhead while retaining pixel-level details.
3) Through the above improvements, this paper aims to construct a high-precision, high-robustness book packaging defect detection model to address the precision bottleneck of existing methods in complex scenarios.

The organization of this paper is as follows: Section 3 offers a detailed description of the proposed method and its enhancements. This is followed by a comprehensive experimental analysis in Section 4 to evaluate model performance and validate the improvements. Section 5 provides a discussion of the results, and finally, Section 6 presents the concluding remarks.

## 3. Methods

**Fig 1:**
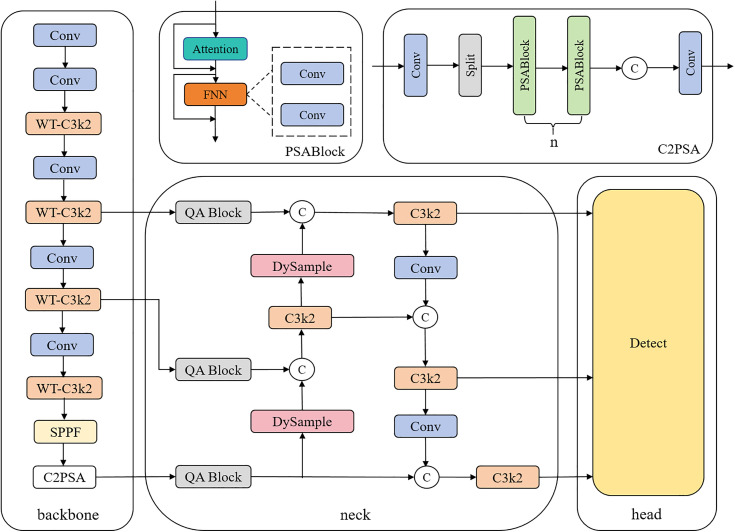
Merge-YOLO network architecture.

This paper proposes a Merge-YOLO network model based on the basic framework of YOLO11, which is designed for defect detection in bookstore logistics packaging. The overall architecture is shown in Fig 1. The model mainly consists of a backbone, neck, and head. The backbone network is used to extract multi-level features of defects, the neck network is used to fuse multi-scale features, and the head is used to generate predictive features for defect classification and detection.

In the backbone network, we use the WT-C3k2 module to replace the C3k2 module, separate low-frequency and high-frequency features through multi-level wavelet decomposition, and use different convolution kernels to improve detection precision. Before performing feature fusion in the neck region, the P3, P4, and P5 output features extracted from the backbone network are input into the QA Transformer Blocks. Through learnable transformation matrices, adaptive quadrilateral windows are generated to enhance the ability to capture multi-morphological defects (irregular damage, tilted scratches), thereby improving the scale adaptability of features at different levels (P3-P5). Additionally, DySample is used to replace the nearest-neighbor interpolation upscaling module in YOLOv11. By dynamically adjusting the scaling ratio through adaptive scope factors and combining Pixel Shuffle operations, pixel-level fine-grained upscaling is achieved.

### 3.1. Wavelet feature extraction module

YOLOv11 is a target detection method based on convolutional neural networks (CNNs), and its performance largely depends on the design of the convolutional layers. However, as the network depth increases, it integrates information by gradually reducing the spatial resolution and expanding the channel dimension, a process that may result in the loss of some semantic details. Although this design has little impact on the detection performance of high-resolution images, in real-time defect detection tasks, the input image resolution is usually low, and the pixel information itself is limited. The spatial features of small defects may be severely compressed after the first downsampling, making it difficult for subsequent layers to capture effective information. This leads to a decrease in detection precision.

Based on the above analysis, this paper utilizes the WTConv module, which the author has verified to be more effective at processing low-frequency and high-frequency information in images, significantly increasing the receptive field without causing excessive parameter increases. Therefore, we consider integrating WTConv into the C3k2 module of YOLOv11 to enhance the network’s feature extraction capabilities. WTConv utilizes Haar wavelets, though other wavelet bases can be employed despite increased computational costs. This is because our model is designed for real-time detection in logistics scenarios. The Haar wavelet is the simplest to compute among all wavelet bases, involving only basic addition and shift operations. This offers significant advantages in hardware deployment and inference speed compared to more complex wavelet bases requiring floating-point multiplication and longer filters. This computational lightness is crucial for ensuring our model’s overall high frame rate of 135 FPS.

If WTConv is directly integrated into the C3k2 module, although it can enhance the model’s ability to capture low-frequency features, since C3k2 already has strong feature extraction capabilities, direct integration may introduce additional computational overhead, preventing the full exploitation of WTConv’s advantages. Therefore, we propose the WT-C3k2 module, whose specific steps are as follows:

Given an input image X , the one-dimensional Haar wavelet transform decomposes the image into low-frequency and high-frequency components for each spatial dimension through deep convolution and downsampling. The two-dimensional Haar wavelet transform uses the following four sets of filters for depth convolution:


$$f_{LL}=\frac{\text{1}}{\text{2}}\left[\begin{array}{cc} {}*20c1 & 1\\ 1 & 1 \end{array}\right],f_{LH}=\frac{\text{1}}{\text{2}}\left[\begin{array}{cc} {}*20c1 & -1\\ 1 & -1 \end{array}\right],f_{HL}=\frac{\text{1}}{\text{2}}\left[\begin{array}{cc} {}*20c1 & 1\\ -1 & -1 \end{array}\right],f_{HH}=\frac{\text{1}}{\text{2}}\left[\begin{array}{cc} {}*20c1 & -1\\ -1 & 1 \end{array}\right]$$ (1)

where $f_{LL}$ is a low-pass filter, and $f_{LH}$, $f_{HL}$ and $f_{HH}$ are a set of high-pass filters. Each set of filters decomposes the image into low-frequency component $X_{LL}$ and high-frequency component $X_{LH}$, $X_{HL}$, $X_{HH}$:


$$\left[X_{LL},X_{LH},X_{HL},X_{HH}]=Conv([f_{LL},f_{LH},f_{HL},f_{HH}],X\right)$$ (2)

After decomposing the defect image into low-frequency components (reflecting the overall structure and contours of the image) and high-frequency components (capturing detailed information such as edges and textures), a recursive approach is employed to perform multi-level wavelet decomposition on the low-frequency components. By repeatedly applying the wavelet transform, the low-frequency components are further decomposed into low-frequency and high-frequency components:


$$X_{LL}^{\left(i\right)},X_{LH}^{\left(i\right)},X_{HL}^{\left(i\right)},X_{HH}^{\left(i\right)}=WT(X_{LL}^{\left(i-1\right)})$$ (3)

Then, by performing convolution operations on each frequency component separately (using a larger convolution kernel to process low-frequency components and a smaller convolution kernel to process high-frequency components), a larger receptive field can be obtained. The convolved frequency components are merged into a feature map Y through inverse wavelet transformation. The specific calculation formula is shown in Equation (4). After feature fusion, we retain the original bottleneck structure for feature compression and information processing. This layer further compresses and adjusts information at different frequencies, reducing redundant features while enhancing the expressive power of key features. The bottleneck layer is implemented through 1×1 convolution, reducing the dimension of the feature map and improving computational efficiency.


Y=IWT(Conv(W,WT(X))) (4)

**Fig 2:**
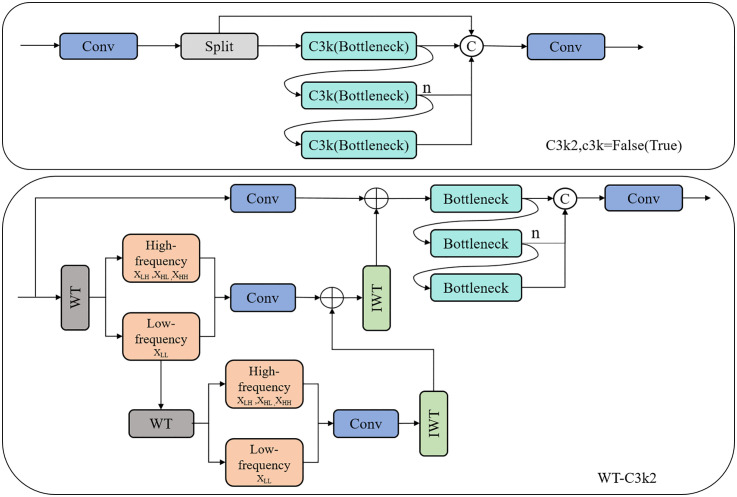
WT-C3k2 module and C3k2 module structure.

The WT-C3k2 module structure is shown in Fig 2. In the defect detection scenario of bookstore logistics packaging, the C3k2 module provides powerful feature extraction capabilities by combining variable convolution kernels and channel separation strategies. The WTConv module, with its expanded receptive field and ability to process different frequency features, not only focuses on detecting small defects, but also grasps the overall structural information of logistics packaging defects from a global perspective, identifying fine structures hidden in the overall features. By combining the advantages of C3k2 and WTConv, the WT-C3k2 module can accurately identify defects in packaging images captured in different storage environments (such as strong light, weak light, backlight, etc.). A balance has been achieved between capturing local defect features and overall structural information. This is crucial for improving the detection precision of small defects and overall detection in bookstore logistics packaging, enabling the system to more accurately detect subtle defects that are easily overlooked and making the entire detection model more stable and reliable.

### 3.2. Quadrilateral window attention module (QA Block)

Attention mechanisms have become one of the key technologies for improving model performance. In the Visual Transformer (ViT) architecture, it can focus on key parts of an image to improve task performance, but the problem with ViT is that its computational complexity is quadratic to the size of the input image. To address this issue, Swin Transformer reduces computational complexity to linear by using local region attention. However, its fixed window size limits its adaptability to different targets. To address these issues, Qiming Zhang et al. proposed the Quadrilateral Attention (QA Transformer). By using a learnable quadrilateral regression module to predict the transformation matrix, the model converts square windows into arbitrary quadrilaterals, directly learning adaptive configurations from the data. This breaks through window limitations and enhances the model’s adaptability to targets of various sizes, shapes, and orientations. In our work, we improved the QA Block and applied QA modules to different levels of features (P3-P5) from Backbone to generate quadrilateral attention adapted to their respective scales. By leveraging its advantages in processing targets of different sizes and shapes, we improved the model’s performance.

The input image is processed by the backbone network to generate feature map $X\in {\mathbb{R}}^{H\times W\times C}$. The feature map is divided into multiple basic windows $X=\{X_{i}|i=1,2,...,\frac{H\cdot W}{w^{2}}\}$ of size w×w. The features within each window are obtained through linear mapping of Q, K, and V. To generate quadrilaterals, QA Block uses a quadrilateral prediction module that predicts a parameterized transformation matrix for each base window. The predicted parameter $t\in {\mathbb{R}}^{9}$ is generated by a network consisting of an average pooling layer, a LeakyReLU activation layer, and a 1×1 convolution layer:


$$t=Conv\circ LeakyReLU\circ AveragePool(X_{w})$$ (5)

Using the output parameter t, the QA Block generates multiple basic transformation matrices, including scaling transformation $T_{s}$, shearing transformation $T_{h}$, rotation transformation $T_{r}$, translation transformation $T_{t}$, and projection transformation $T_{p}$. These matrices are generated using the following formulas:


$$T_{s}=\left[\begin{array}{ccc} {}*20ct_{1}+1 & 0 & 0\\ 0 & t_{2}+1 & 0\\ 0 & 0 & 1 \end{array}\right],T_{h}=\left[\begin{array}{ccc} {}*20c1 & t_{3} & 0\\ t_{4} & 1 & 0\\ 0 & 0 & 1 \end{array}\right],T_{r}=\left[\begin{array}{ccc} {}*20c\cos t_{5} & -\sin t_{5} & 0\\ \sin t_{5} & \cos t_{5} & 0\\ 0 & 0 & 1 \end{array}\right]$$ (6)

$$T_{t}=\left[\begin{array}{ccc} {}*20c1 & 0 & \frac{W}{w}t_{6}\\ 0 & 1 & \frac{H}{w}t_{7}\\ 0 & 0 & 1 \end{array}\right],T_{p}=\left[\begin{array}{ccc} {}*20c1 & 0 & 0\\ 0 & 1 & 0\\ t_{8} & t_{9} & 1 \end{array}\right]$$ (7)

All basic transformation matrices are obtained through sequential multiplication to obtain the final projection transformation matrix T:


$$T=T_{s}\cdot T_{h}\cdot T_{r}\cdot T_{t}\cdot T_{p}$$ (8)

Using the projection transformation matrix T, for each point coordinate $\left(x_{r},y_{r}\right)$ in the base window, find its new coordinate in the target quadrilateral through projection transformation and normalize it to the final coordinate:


$$\left[\begin{array}{c} {}*20cx^{\prime }\\ y^{\prime }\\ z^{\prime } \end{array}\right]=T\cdot \left[\begin{array}{c} {}*20cx_{r}\\ y_{r}\\ 1 \end{array}\right]$$ (9)

$$x_{f}=\frac{x^{\prime }}{z^{\prime }},\;\;\;\;y_{f}=\frac{y^{\prime }}{z^{\prime }}$$ (10)

After obtaining the target quadrilateral and the coordinates of each point within the quadrilateral, the feature K and V of the key and value are sampled in the feature map X using the bilinear interpolation method. The output feature F is obtained by performing self-attention calculation using the sampled K and V and the original Q:


$$F=soft\max(QK^{T})V$$ (11)

Specifically, if the coordinates fall outside the feature map, we abandon the zero-padding method used in the original QA method and instead use mirror padding. This is because when the quadrilateral exceeds the boundary of the feature map, directly assigning the sampling point to zero will introduce noise when calculating attention. Furthermore, when part of the target object is located at the edge of the feature map, using zero-padding may cause the model to lose critical information in these regions, whereas mirror-padding preserves contextual information near the boundaries.

Defects in book logistics packaging (such as cracks and scratches) are mostly elongated or linear in structure and tend to be concentrated in horizontal or vertical directions. In order to better match the shape of these defects, we designed length and width constraints $R_{\left(x\right)}$ and direction constraints $R_{\left(p\right)}$. The length and width constraint $R_{\left(x\right)}$ is used to force the quadrilateral to converge toward an elongated structure. The direction constraint $R_{\left(p\right)}$ is used to concentrate the rotation angle t at 0 or π/2. The defined penalty terms are as follows:


$$R_{\left(x\right)}=\sum \lambda \cdot \max(0,3-\frac{\max(w_{i}^{\prime },h_{i}^{\prime })}{\min(w_{i}^{\prime },h_{i}^{\prime })})$$ (12)

$$R_{\left(p\right)}=\sum \lambda \cdot (|\sin(2t_{5})|+|\cos(2t_{5})-1|)$$ (13)

where λ is the weighting coefficient used to adjust the relative importance of the penalty term in the total loss function. It can be optimized and determined within the range of 0.01–0.1 in experiments. $w_{i}^{\prime }$ and $h_{i}^{\prime }$ are the width and height of the transformed quadrilateral, which can be derived from $T_{s}$ and $T_{t}$. If the aspect ratio is less than 3, it indicates that the aspect ratio of the quadrilateral does not meet the preset rectangular requirements, resulting in a non-zero value, which prompts the model to adjust the transformation parameters. If the aspect ratio is greater than or equal to 3, the penalty term is 0.

Through these steps, QA Block can flexibly generate quadrilaterals that adapt to various targets, enhance feature representation capabilities, and effectively capture contextual information from objects of different shapes, sizes, and directions.

### 3.3. Dynamic Up-sampling (DySample)

The first two improvements slowed down the model’s inference speed by 16.1 ms. To balance speed and precision, we introduced the DySample sampler in the neck to replace the original upsampling module.

The upsampling module in YOLOv11 uses nearest-neighbor interpolation, which may lose important details when enlarging the resolution feature map, introducing issues such as blurring effects and increased computational overhead [40].

DySample is an ultra-lightweight and efficient dynamic upscaler designed to analyze image features to determine the complexity of each region, and then apply different upscaling rates to each region. Pixel-level processing is performed using flexible and variable sampling positions. This method not only reduces unnecessary computational overhead, but also enables the network to transmit information more smoothly between different scales. Maximizes image quality while maintaining computational efficiency, reducing model inference latency, memory usage, and floating point operations (FLOPs). We use the variant DySample-S+ from the DySample series. DySample-S+ uses a dynamic scope factor to dynamically adjust the scaling factor based on the input feature map, and then enlarges the feature map through a pixel shuffle operation. Similarly, the generated feature map *G* and the dynamic offset feature map *O* are added together to obtain the final output 𝑆. Dynamic factors can adaptively adjust the scaling scale of different inputs, making them particularly suitable for processing images with multi-scale features or large resolution variations. This mechanism can better capture detailed information at different scales, improving the flexibility and adaptability of the model.

**Fig 3:**
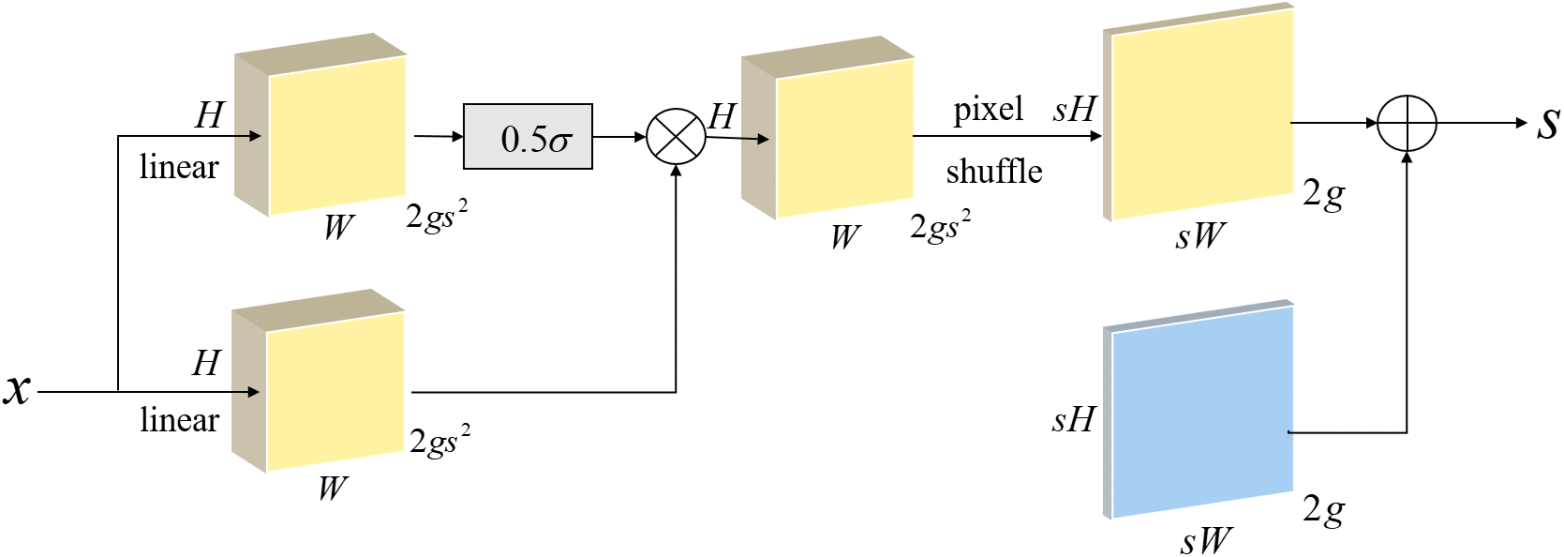
DySample dynamic point sampler.

The structure of the DySample dynamic point sampler is shown in Fig 3:

## 4. Experiments

### 4.1. Experimental environment

To ensure the rigor of the experiment, we conducted experiments under the same server configuration to strictly evaluate the effectiveness of the improved algorithm. The experimental environment configuration is as follows: the operating system is Windows 11, paired with NVIDIA GeForce RTX 4060 and Intel Core i9-12900HX. The deep learning framework is PyTorch 1.11, Python version is 3.9, and CUDA version is 11.6.

In the experiment, the optimizer selected stochastic gradient descent (SGD), set the input image size to 640 × 640, the total training cycle of the model to 100, the number of training samples in each batch to 16, and the initial learning rate to 0.01.

The relevant configurations for this experiment are shown in Table 1.

**Table 1:** Configurations related to this experiment.

Name	Configure
Operating System	Windows 11
CPU	Intel Core i9-12900HX
GPU	NVIDIA GeForce RTX 4060
RAM	16GB
IDE	PyCharm2020
Deep Learning Frameworks	PyTorch1.11
Programming Language	python3.9
Additional package	CUDA11.6、CUDnn8.0.4、OpenCV4.6.0.6等

### 4.2. Dataset

To validate the effectiveness of the proposed method in target defect detection tasks while addressing practical production challenges, we conducted experiments on a dataset encompassing three primary defect types: tears, scratches, and damage. The dataset comprised 926 tear images, 778 scratch images, and 1120 damage images, totaling 2824 images. These logistics packaging defects exhibit significant scale diversity. For instance, some defects appear as elongated scratches or indentations, while others manifest as localized dents or large-area stains. The substantial variations in morphology and size pose significant challenges to traditional detection models, which often struggle to accurately identify these defect types simultaneously.

**Fig 4:**
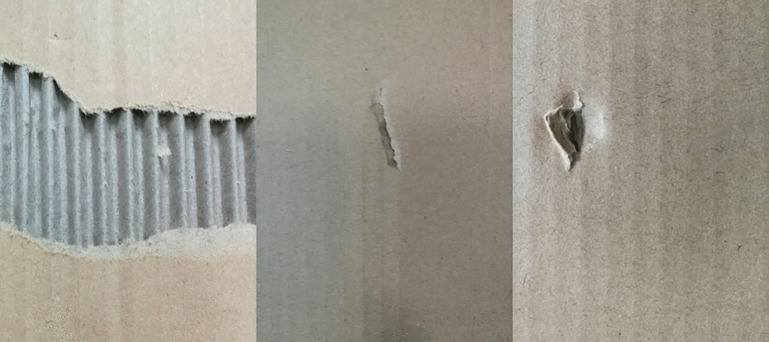
Illustrations of various defects (from left to right: tears, scratches, and damage).

All images were standardized to a size of 640 × 640. The data was then divided according to an 8:1:1 ratio, resulting in a training set of 2,260 images, a validation set of 282 images, and a test set of 282 images. This division ensures that the model can learn from sufficient training data while also enabling performance evaluation on independent validation and test sets. All defect images were annotated using the Labelimg software, with annotations made as small as possible, and the results were stored in a txt file to record the locations of the defects. Examples of various defect types are shown in Fig 4.

### 4.3. Evaluation criteria

This study uses parameters and FLOPs as evaluation indicators for model size and computational complexity. Model performance is evaluated using precision P, recall R, mean average precision mAP, and frames per second FPS.

Precision: The ratio of true positives (TP) to all samples predicted as positive (TP + FP). That is, how many of the samples predicted as defective are actually defective.

Recall: The ratio of true positives (TP) to all samples with actual defects (TP + FN). That is, how many of the samples that are actually defective are predicted to be abnormal.

mAP (Mean Average Precision): The average precision of all categories, which is an important indicator for evaluating the overall detection performance of a model.

FPS refers to the detection speed of the model, i.e., the number of images that can be processed per second.

The calculation formulas for each evaluation indicator are as follows:


$$P=\frac{TP}{TP+FP}$$ (14)

$$R=\frac{TP}{TP+FN}$$ (15)

$$mAP=\frac{1}{N_{c}}\underset{i=1}{\overset{N_{c}}{\sum }}\;\;AP_{i}$$ (16)

$$F_{FPS}=\frac{N}{T}$$ (17)

where TP represents the number of samples correctly predicted as positive; FP represents the number of samples incorrectly predicted as positive; FN represents the number of samples that are actually positive but incorrectly detected as negative; $N_{c}$ represents the number of defect categories; N represents the number of images detected; T represents the total detection time.

### 4.4. Experimental results and analysis

#### 4.4.1. Comparison experiment.

This study improves upon the YOLOv11 base model. To validate the effectiveness of the proposed Merge-YOLO algorithm, we selected eight models for comparison: YOLOv5, YOLOv6, YOLOv7, YOLOv8, YOLOv9, YOLOv10, Faster R-CNN, and SSD.

YOLOv5, YOLOv6, YOLOv7, YOLOv8, YOLOv9, YOLOv10, and YOLOv11 are all part of the same series of models. They are based on the same core design philosophy, with differences between versions primarily lying in detailed improvements (such as backbone network optimization, loss function design, and training strategy adjustments). By comparing with models within the same series, experiments can focus on the improvements themselves rather than differences in architectural design. In addition to comparing models within the same series, we also selected Faster R-CNN and SSD for comparison experiments. As representative algorithms for two-stage and one-stage object detection, respectively, they are widely applied in various scenarios due to their high-quality detection results and efficient inference speeds, further validating the universality of the improvements. By comparing them, we can better evaluate the performance of our model. The performance metrics of each algorithm are shown in Table 2.

**Table 2:** comparative test results.

Algorithm	Precision (%)	Recall (%)	mAP @0.5	FPS	Parameter (M)	FLOPs (G)
YOLOv5 [41]	92.5	91.7	93.1	83.1	7.2	16.1
YOLOv6 [42]	93.4	92.7	93.9	83.2	17.2	16.3
YOLOv7 [43]	90.9	87.2	93.3	97.1	**6.03**	13.7
YOLOv8 [44]	95.3	86.6	91.2	103.1	11.2	28.2
YOLOv9 [45]	94.4	82.8	89.6	125	7.2	26.7
YOLOv10 [46]	88.7	83.4	89.3	129	7.2	26.7
YOLOv11 [47]	93.7	85	91.1	112	9.4	21.5
SSD [48]	84.98	86	86.06	52.3	62.74	26.29
Faster R-CNN [49]	85.7	85.2	87.05	40	137	370.2
Merge-YOLO	**95.8**	**93.6**	**94.1**	**135**	9.3	**13**

Precision: The improved Merge-YOLO algorithm achieves a precision of 95.8%, with an extremely high proportion of correctly predicted results in detection outcomes. This represents a 2.1% improvement in precision compared to the original YOLOv11 algorithm. YOLOv8 achieves 95.3%, YOLOv6 achieves 93.4%, and YOLOv11 achieves 93.7%. In traditional algorithms, Faster R-CNN achieves an precision of 85.7%, and SSD achieves 84.98%, both significantly lower than the YOLO series and our algorithm, indicating that the new algorithm has a clear advantage in accurately identifying targets.

Recall: The Merge-YOLO algorithm achieved 93.6%, effectively detecting most targets, an improvement of 8.6% over the original YOLOv11 algorithm. YOLOv6 and YOLOv5 followed closely behind with 92.7% and 91.7%, respectively. SSD and Faster R-CNN achieved recall rates of 86% and 85.2%, respectively, placing them in the middle range.

mAP@0.5: The average precision of the Merge-YOLO algorithm reached 94.1%, an improvement of 3%. Most YOLO versions fluctuate around 90% mAP@0.5, 93.9% for YOLOv6, 93.1% for YOLOv5, and 93.3% for YOLOv7. Among the traditional algorithms, Faster R – CNN is 87.05% and SSD is 86.06%, and the new algorithm has obvious advantages in this indicator and is better suited to target detection tasks in complex scenarios.

FPS: The Merge-YOLO algorithm achieves 135 FPS, offering faster detection speeds and enhanced real-time performance. YOLOv10 follows closely with 129 FPS, YOLOv9 with 125 FPS, and YOLOv8 at 103.1 FPS. In contrast, Faster R-CNN achieves only 40 FPS, significantly lagging behind the YOLO series and our algorithm in detection speed, making it unsuitable for applications requiring high real-time performance.

Parameter: YOLOv7 has the fewest parameters, at 6.03 million, while the Merge-YOLO algorithm has 9.3 million. Compared to the original YOLOv11 model, there has been no significant change in the number of parameters. The parameter counts for YOLOv5, YOLOv9, and YOLOv10 are similar. Faster R-CNN has 137 million parameters, while SSD has 62.74 million. Compared to the YOLO series, these models may be more suitable for scenarios with less stringent performance requirements.

FLOPs: The Merge-YOLO algorithm has a FLOPs value of 13G, which is relatively low. YOLOv7 has a FLOPs value of 13.7G, and YOLOv5 has a FLOPs value of 16.1G. Faster R-CNN reaches 370.2G, and SSD has a FLOPs value of 26.29G. This indicates that the Merge-YOLO algorithm and some YOLO algorithms have low computational complexity and require minimal computational resources during runtime.

In summary, the improved Merge-YOLO algorithm performs excellently in key metrics such as precision, recall rate, mAP, and FPS, with moderate parameter counts and computational requirements. Compared to traditional algorithms and some YOLO series algorithms, it demonstrates better overall performance in object detection tasks.

Below, we analyze the reasons for the high and low precision of these algorithms.

The YOLO series of algorithms generally achieve high precision in logistics packaging defect detection. This performance advantage stems from their powerful deep network architectures, which excel at learning abstract and discriminative features from large datasets of defect images. This enhanced feature representation enables more accurate identification of various defect types. Furthermore, YOLO’s multi-scale detection mechanism allows it to effectively handle defects of different sizes, maintaining high detection precision for large, medium, and small defects—including damage, scratches, and tears.

Due to the relatively simple architecture of its feature extraction network, the SSD algorithm has a limited capacity to learn subtle and complex defect features. This limitation is evident in its difficulty in accurately detecting shallow, fine scratches, which consequently constrains its overall detection precision.

The Faster R-CNN algorithm is computationally expensive and structurally complex. Moreover, whether it employs VGGNet or ResNet as its backbone, the feature maps it extracts are single-layer and of relatively low resolution. These limitations collectively lead to its suboptimal precision in logistics packaging defect detection.

Table 3 presents the detection results of the YOLOv11 and Merge-YOLO models for three defect types. The findings indicate that the YOLOv11 model achieves a relatively low recall rate for “broken” defects, leading to a considerable number of missed detections. In comparison, the Merge-YOLO model demonstrates superior performance across both precision and recall metrics. Specifically, it outperforms YOLOv11 in precision by 1.2% for “fold,” 3.5% for “scratch,” and 1.7% for “broken” defects. More notably, in terms of recall, Merge-YOLO surpasses YOLOv11 by 5% for “fold,” 5% for “scratch,” and 16% for “broken” defects. These results confirm that the Merge-YOLO model can identify book packaging defects more accurately and comprehensively in practical scenarios, exhibiting higher overall detection efficiency.

**Table 3:** Detection results of the improved and original algorithms on different types of defects.

Algorithm	Precision (%) / Recall (%)		
	fold	scratch	broken
ours	96.2%/ 95%	95.5%/ 92%	95.7%/ 94%
YOLOv11	95%/ 90%	92%/ 87%	94%/ 78%

#### 4.4.2. Visual analysis.

To visually demonstrate the superiority of the algorithm described in this paper, the figure below shows the visualization results of the original YOLOv11 algorithm and the improved Merge-YOLO algorithm on the defect dataset. The red boxes indicate missed defects. By comparing the performance differences between the two models in their detection results, we explore the effectiveness of the improvements.

**Fig 5:**
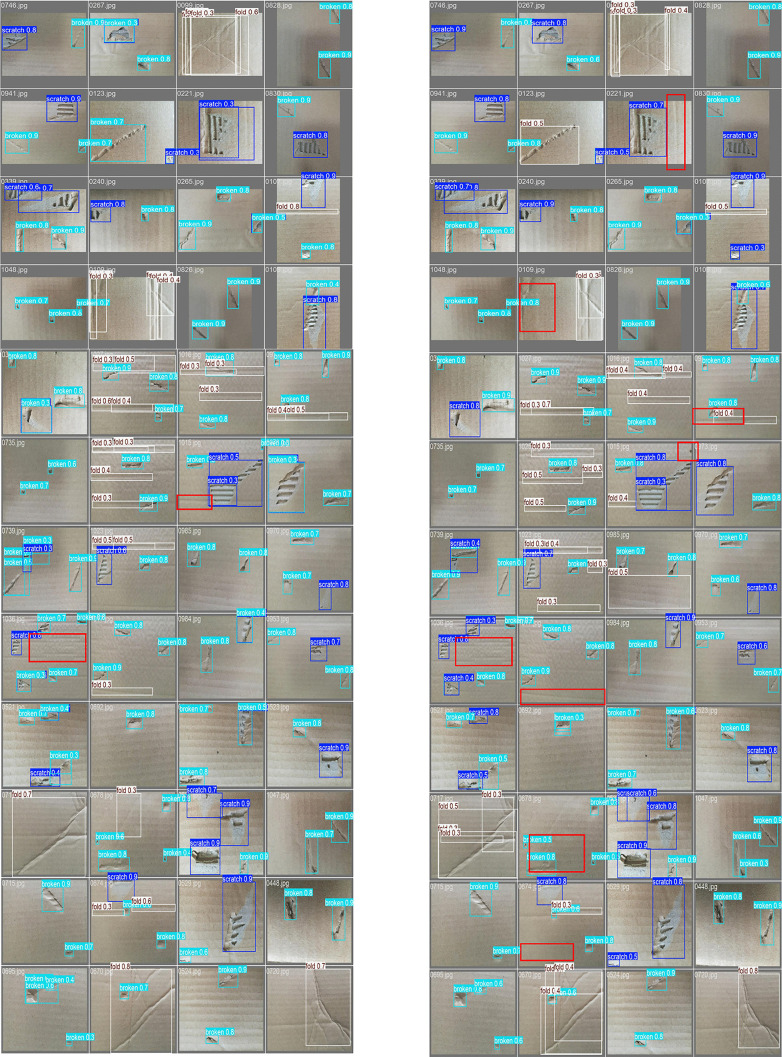
Visual comparison of Merge-YOLO and YOLOv11.

**Fig 6:**
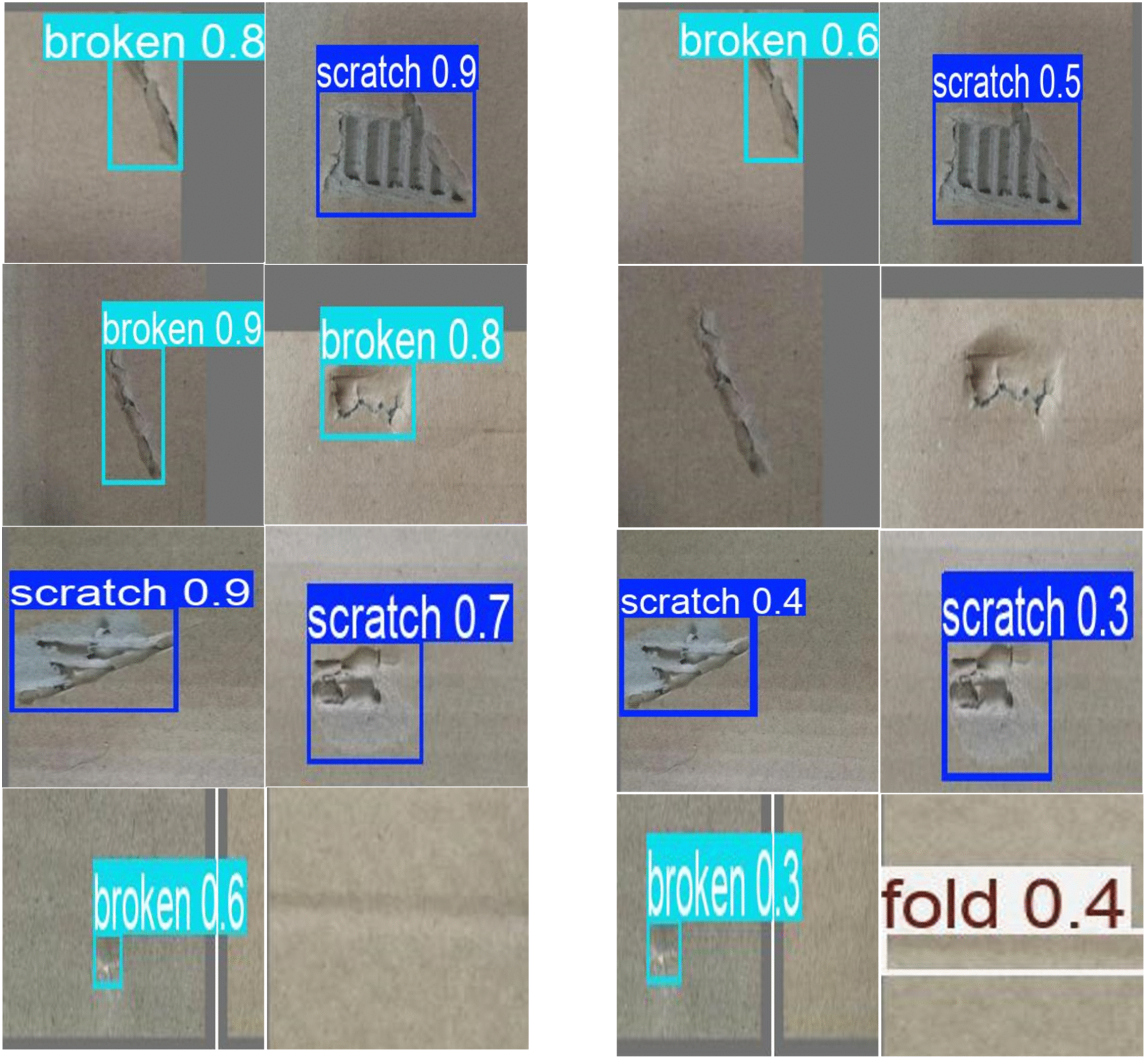
Comparison of Merge-YOLO and YOLOv11 with amplified defects.

As shown in Figs 5 and 6, the YOLOv11 model has multiple red missed detection areas, which are scattered across different locations and scales. For example, in some images containing dense small objects, some small targets are not covered by the detection box. At the same time, in cases where the contrast between the target defect and the background was low, there were also many missed detections. Merge-YOLO significantly reduced the number of missed red areas and had a high detection confidence for all types of defects. In scenarios where small targets were easily missed, the improved model successfully detected more small targets and had a significantly wider detection range. For targets with low contrast against the background and irregular shapes, false negatives have also been greatly improved.

Although this visualization primarily focuses on detection results, we analyzed the causes of this phenomenon from the perspective of model improvement: the original YOLOv11 model has limited ability to capture features of small targets during feature extraction, making it difficult to accurately describe defects in small targets; after multi-layer downsampling, the features of small targets may become overly abstract and blurred, losing some key position and detail information, all of which may lead to issues with detection results. The improved model enhances feature extraction capabilities for small objects by modifying the C3k2 module using WTConv and applying quadrilateral attention, enabling the model to learn more object features during training and reduce false negatives.

#### 4.4.3. Ablation experiment.

We mainly optimized the YOLOv11 base model in three ways: introducing WTConv wavelet convolution to reconstruct the C3k2 module, using quadrilateral attention (QA) to optimize the neck, and introducing DySample dynamic point sampler. To assess the impact of different improvement measures on network performance, we analyzed the effectiveness of each module through ablation experiments, with “√” indicating that the corresponding improvement strategy was used in the model. The experimental results are shown in Table 4.

**Table 4:** Ablation experiments were conducted to analyze the effectiveness of the different improvement strategies in this paper.

Mould	WT-C3k2	QA Block	DySample	Precision (%)	FPS	mAP @0.5	Parameter (M)
YOLO11				93.7	112	91.1	9.4
1	√			94.8	96	93.2	9.7
2	√	√		95.5	96	93.9	9.7
3	√	√	√	95.8	135	94.1	9.3

From the table, we can see that after replacing the C3k2 module with the WTConv module, Precision increased from 93.7% to 94.8% of YOLO11, increased from 91.1% to 93.2% of mAP@0.5, FPS decreased from 112 to 96, and the number of parameters increased from 9.4M to 9.7M. On the basis of the existing WTConv module, the QA module is added, and the Precision is further increased to 95.5%, mAP@0.5 to 93.9%, the FPS remains unchanged, and the number of parameters is still 9.7M. When three modules are introduced into the YOLOv11 model at the same time, Precision increases to 95.8%, mAP@0.5 to 94.1%, FPS increases significantly to 135, and the number of parameters drops to 9.3M.

Based on the results of the ablation experiment, it can be seen that WTConv sacrifices a small amount of inference speed and increases the amount of parameters, but all within acceptable limits. The QA module continues to improve Precision and mAP@0.5 without additional negative impact on speed and parameter volume. The addition of DySample optimizes speed and parameter volume while improving performance. Overall, the proposed model focuses on the defects of book logistics packaging, and the use of WTConv, QA and DySample modules can achieve a good balance between precision, speed and parameter quantity, and can detect target defects more accurately.

## 5. Discussion

The Merge-YOLO model proposed in this paper demonstrates significant advantages in the detection of defects in book logistics packaging. Merge-YOLO can be deployed on edge computing devices along warehouse sorting lines. It captures real-time images of book packaging via cameras, with model inference results directly fed back to the control system to enable automatic sorting and alerts for defective packaging. The system supports parallel processing across multiple cameras, meeting real-time inspection demands in high-throughput logistics scenarios. Comparison of experimental results shows that Merge-YOLO performs excellently in key metrics such as precision, recall rate, mAP, and FPS. This is mainly due to the WT-C3k2 module expanding the receptive field through multi-level wavelet decomposition, combined with the cross-level feature adaptive fusion of the QA Transformer, which solves the problem of small target features being easily lost. In addition, ablation experiments further verify the effectiveness of different modules.

Although in actual warehousing and logistics scenarios, the Merge-YOLO model achieves an average detection precision of 95.8% for three types of defects (tears, scratches, and damage), with a false negative rate 8.6% lower than YOLOv11. Its detection speed also meets real-time requirements, significantly improving packaging quality control efficiency. However, the model still has the following limitations: the current dataset only includes three major defect types and does not cover niche defect types such as tape misalignment and printing contamination, requiring further expansion of data diversity; Although the number of parameters is slightly reduced compared to YOL11, further compression of the model size is still required for deployment on edge devices; Although the model performs excellently in experimental environments, it may face more complex lighting changes and background interference in actual warehouse scenarios, necessitating further validation of its robustness in real-world applications.

## 6. Conclusions

This paper addresses key challenges in defect detection for book logistics packaging by proposing Merge-YOLO, an enhanced model based on YOLOv11. The model integrates a wavelet transform feature extraction module (WT-C3k2), a quadrilateral window attention mechanism (QA Transformer Block), and a dynamic upsampling component (DySample), leading to significant improvements in both precision and efficiency. In subsequent experiments, Merge-YOLO was compared against mainstream detection models, including YOLOv5–YOLOv10, Faster R-CNN, and SSD. We further conducted an in-depth analysis of the factors contributing to performance differences. Experimental results demonstrate that Merge-YOLO outperforms existing methods in terms of Precision, Recall, mAP@0.5, and FPS. It effectively mitigates issues such as missed detection of small objects, insufficient robustness in complex environments, and the challenge of meeting real-time requirements—common limitations in book packaging defect detection. The model thus fulfills the practical demands of industrial deployment with considerable application value. Future work will focus on dataset expansion, model lightweighting, and validation in real-world scenarios to further promote the industrial adoption of this approach.
